# Endothelial cells differentially sense laminar and disturbed flows by altering the lipid order of their plasma and mitochondrial membranes

**DOI:** 10.1152/ajpcell.00393.2023

**Published:** 2023-11-06

**Authors:** Kimiko Yamamoto, Yuji Shimogonya, Ryohei Maeno, Kenshiroh Kawabe, Joji Ando

**Affiliations:** ^1^Laboratory of System Physiology, Department of Biomedical Engineering, Graduate School of Medicine, The University of Tokyo, Tokyo, Japan; ^2^Department of Mechanical Engineering, College of Engineering, Nihon University, Koriyama, Japan; ^3^Division of Vascular Surgery, Department of Surgery, Graduate School of Medicine, The University of Tokyo, Tokyo, Japan; ^4^Laboratory of Biomedical Engineering, School of Medicine, Dokkyo Medical University, Tochigi, Japan

**Keywords:** disturbed flow, endothelial cells, mitochondria, membrane lipid order, shear stress

## Abstract

Endothelial cells (ECs) experience two different blood flow patterns: laminar and disturbed flow. Their responses to laminar flow contribute to vascular homeostasis, whereas their responses to disturbed flow result in EC dysfunction and vascular diseases. However, it remains unclear how ECs differentially sense laminar and disturbed flow and trigger signaling that elicits different responses. Here, we showed that ECs differentially sense laminar and disturbed flows by altering the lipid order of their plasma and mitochondrial membranes in opposite directions. This results in distinct changes in mitochondrial function, namely, increased adenosine triphosphate (ATP) production for laminar flow and increased hydrogen peroxide (H_2_O_2_) release for disturbed flow, leading to ATP- and H_2_O_2_-mediated signaling, respectively. When cultured human aortic ECs were subjected to laminar or disturbed flow in flow-loading devices, the lipid order of their plasma membranes immediately decreased in response to laminar flow and increased in response to disturbed flow. Laminar flow also decreased the lipid order of mitochondrial membranes and increased mitochondrial ATP production. In contrast, disturbed flow increased the lipid order of mitochondrial membranes and increased the release of H_2_O_2_ from the mitochondria. The addition of cholesterol to the cells increased the lipid order of both membranes and abrogated laminar flow-induced ATP production, while treatment of the cells with a cholesterol-depleting reagent, methyl-β cyclodextrin, decreased the lipid order of both membranes and abolished disturbed flow-induced H_2_O_2_ release, indicating that changes in the membrane lipid order and/or cholesterol content are closely linked to flow-induced changes in mitochondrial functions.

**NEW & NOTEWORTHY** How vascular endothelial cells (ECs) differentially sense laminar and disturbed flows and trigger intracellular signaling remains unclear. Here, we show that EC plasma membranes act as mechanosensors to discriminate between laminar and disturbed flows by undergoing opposite changes in their lipid order. Similar lipid order changes occur simultaneously in the mitochondrial membranes, which are linked to changes in mitochondrial function, that is, increased ATP production for laminar flow and increased H_2_O_2_ release for disturbed flow.

## Graphical abstract

**Figure:**
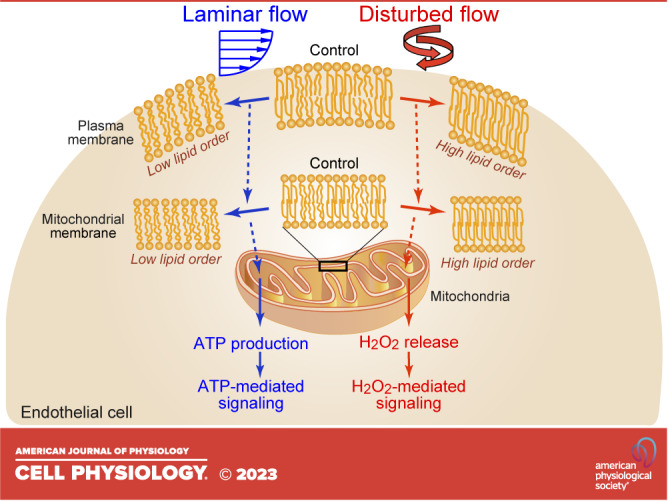

## INTRODUCTION

Vascular endothelial cells (ECs) sense shear stress generated by flowing blood and transmit this information into the cell interior, leading to EC responses involving changes in morphology, function, and gene expression (1). ECs can experience two different flow patterns depending on their location in vascular trees. Unidirectional laminar flow occurs in straight conduit arteries, whereas disturbed flow with unsteady direction and velocity occurs in certain areas of the arterial bends and branches. Many studies have shown that ECs respond differently to laminar flow and disturbed flow and that EC responses to laminar flow contribute to the maintenance of vascular homeostasis, while responses to disturbed flow lead to impaired EC function and the development of vascular diseases such as aneurysms and atherosclerosis (2–4). However, it remains unclear how ECs separately sense laminar and disturbed flows and transmit this information into the cell interior to elicit different EC responses.

EC sensing and signaling of shear stress have been studied extensively, with a focus on laminar flow. Laminar flow has been shown to activate a wide variety of intracellular signaling pathways via membrane molecules, such as ion channels, receptors, adhesion molecules and proteoglycans, cytoskeletons, and membrane microdomains, including caveolae and primary cilia; these pathways resulted in changes in various EC functions (5). Furthermore, the plasma membrane itself has been shown to play an important role in the sensing and signaling of laminar flow (6). EC plasma membranes rapidly respond to laminar flow by altering their physical properties, such as their fluidity and viscosity, leading to the activation of G proteins and mitogen-activated protein kinase (7–10). Our previous study showed that laminar flow immediately altered the physical properties of the EC plasma membrane by decreasing the lipid order, increasing membrane fluidity, and decreasing the membrane cholesterol content, which triggered ATP release into the extracellular space and purinergic-receptor-mediated Ca^2+^ signaling (11–13). This Ca^2+^ signaling plays a crucial role in the control of vascular tone and remodeling through the production of a potent vasodilator, nitric oxide (14). Thus, the sensing and signaling of laminar flow has been elucidated to a large extent; however, that of disturbed flow remains largely unknown.

For some time now, mitochondria have been attracting attention for their function as signaling organelles that transmit information into the cell when the cells are subjected to changes in environmental conditions (15–17). Recent data have shown a direct role of mitochondria in the EC mechanotransduction of fluid shear stress (18, 19). We previously demonstrated that the application of laminar flow to ECs immediately increased mitochondrial ATP production by enhancing oxidative phosphorylation, leading to the above-mentioned ATP release and purinergic Ca^2+^ signaling (13, 20). However, it is not yet known how shear stress acting on the plasma membrane alters the intracellular mitochondrial function. Recently, it has become apparent that mitochondrial functions are regulated not only by the concentration of oxygen and ADP in the matrix and mitochondrial membrane potential but also by the physical properties of the mitochondrial membrane, such as viscosity and lipid order (21). For example, oxidative phosphorylation was shown to be enhanced when the viscosity of the mitochondria inner membrane was decreased by increasing the amount of unsaturated lipids. However, whether flow stimulation of ECs affects the physical properties of mitochondrial membranes has not been investigated to date. The inability to measure the physical properties of mitochondrial membranes has prevented such research, but a new imaging method has recently been developed, making this possible (22).

The present study aimed to elucidate how ECs sense and signal laminar and disturbed flow, focusing on the roles of the plasma membrane and mitochondria. To achieve this purpose, we applied laminar flow or disturbed flow to cultured human aortic ECs (HAECs) in flow-loading devices and analyzed the changes in the lipid order of both plasma membranes and mitochondrial membranes, mitochondrial ATP production, and hydrogen peroxide (H_2_O_2_) release using real-time imaging methods with various optical probes.

## MATERIALS AND METHODS

### Cell Cultures and Treatments

HAECs were purchased from Lonza Group Ltd. (Material number: ml-2535) and were grown in M199 supplemented with 15% fetal bovine serum, 2 mM l-glutamine (Gibco), 50 µg/mL heparin, and 30 µg/mL EC growth factor (Becton Dickinson) in a 1% gelatin-coated tissue culture flask. The cells used in the experiments in this study were in the 7th and 10th passages. All the experiments were approved by the Ethics Committee of the University of Tokyo, Graduate School of Medicine.

Cholesterol reduction was induced by incubating HAECs with 10 mM methyl-β-cyclodextrin (MβCD, Sigma-Aldrich) dissolved in Hanks’ Balanced Salt solution (HBSS, Sigma-Aldrich) for 30 min at 37°C. Cholesterol enrichment was achieved by incubating HAECs with a complex of cholesterol (Sigma-Aldrich) and MβCD (1:7 molar ratio) dissolved in the complete culture medium for 6 h at 37°C. The final concentration of cholesterol was 100 µM. To inhibit the mitochondrial respiratory chain complex I, HAECs were treated with 5 µM rotenone (Sigma-Aldrich) dissolved in HBSS for 10 min at 37°C.

### Flow-Loading Experiments

**Figure 1.**
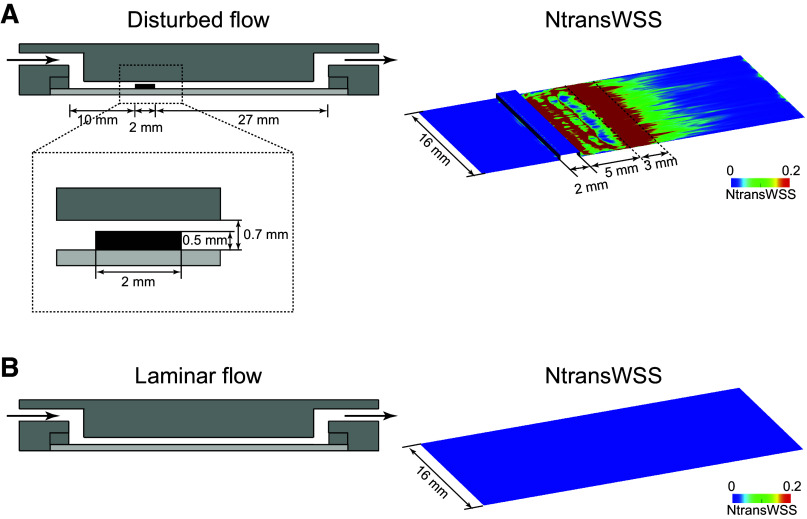
Flow-loading devices. A parallel-plate dynamic flow system was used to apply disturbed flow or laminar flow to cultured ECs. *A*: the flow chamber consisted of a cover glass with cultured ECs on the bottom side and a parallel glass plate held 700 µm apart from the bottom plate and had a motorized height-changeable step near the inlet, which created a disturbed flow downstream of the step. The normalized transverse wall shear stress (NtransWSS) was calculated using computer fluid dynamics (CFD) analysis and used as a metric for disturbed flow. The colors represent different NtransWSS values according to the scale. The cells located 5–8 mm downstream of the step were subjected to disturbed flow with an NtransWSS of 0.2 or more (average of, 0.4) and an average WSS of 20 dynes/cm^2^ by adjusting the step height and flow rate. *B*: in contrast, the cells were exposed to laminar flow with an NtransWSS of zero and a WSS of 15 dyne/cm^2^.

We fabricated a parallel-plate dynamic flow system that produces a disturbed flow that mimics the flow characteristics of human arteries. The flow chamber consisted of a 1%-gelatin-coated cover glass with the cultured cells on one side and a second, parallel, glass plate held 700 µm apart from the first plate by a Teflon gasket (Fig. 1). The medium was perfused using a roller/tube pump, and the entire closed circuit was maintained at exactly 37°C. The flow chamber had a motorized height-changeable step near the inlet, which created a disturbed flow characterized by a recirculation eddy immediately downstream of the step, followed by a region of flow reattachment. As a metric for disturbed flow, we used the normalized transverse wall shear stress (NtransWSS), which was calculated using a computer fluid dynamics (CFD) analysis based on the geometrical parameters of the flow chamber and the viscosity, density, and velocity of the culture medium (23). NtransWSS represents the degree of disturbance in the strength and direction of the shear stress, with a maximum value of 1.0. Since disturbed flow with an NtransWSS ranging from 0.2 to 1.0 has been shown to occur in human carotid bifurcations where atherosclerotic plaques are prone to develop (24), we subjected the cells to a disturbed flow with an NtransWSS of 0.2 or more (average, 0.4) and an average WSS of 20 dynes/cm^2^ by adjusting the step height and flow rate (Fig. 1*A*).

In contrast, a parallel-plate flow chamber without steps was used for experiments in which laminar flow was applied to ECs. The intensity of the shear stress (τ, dynes/cm^2^) acting on the EC layer was calculated from the formula τ = 6µQ/*a*^2^*b*, where µ is the viscosity of the perfusate (poise), Q is the flow volume (mL/s), and *a* and *b* are the cross-sectional dimensions of the flow path (cm). In the present experiments, the cells were exposed to laminar flow with an NtransWSS of zero (Fig. 1*B*) and a WSS of 15 dyne/cm^2^, which was within the range of physiological levels (10–20 dynes/cm^2^) in large to medium-sized human arteries (25).

### CFD Analysis

To analyze the fluid flow and quantify NtransWSS in the flow-loading device, we first generated computational meshes with a resolution of 0.02–0.05 mm and a total element number of 9,268,480 for the geometry model of the device using the Mixed-Element Grid Generator in 3 Dimensions (MEGG3D) (26). The fluid flow in the device was simulated by solving the Navier-Stokes equations using the CFD software OpenFOAM 8 (OpenCFD Ltd.); NtransWSS was then quantified on the bottom of the device. In this simulation, the HBSS fluid was treated as an incompressible Newtonian viscous fluid with a density of 1,010 kg/m^3^ and a viscosity of 0.77 mPa·s, and its flow rate was set to 200 mL/min.

### Imaging of Plasma Membrane Lipid Order

Real-time Laurdan imaging was performed to analyze the lipid order of the plasma membranes, as described previously (11). Lipid order is a physical property of lipid bilayer membranes and depends on the type, distribution, and density of lipids, the orientation and kinetic state of the acyl chains of phospholipids, and the cholesterol content. As the lipid order increases, the cell membrane becomes more rigid and less fluid, while the opposite phenomena occur when the lipid order decreases. Cells labeled with Laurdan dye (Molecular Probes) were placed in mechanical force-loading devices and then mounted on the stage of a confocal laser-scanning microscope (TCS SP2 AOBS, Leica Microsystems) equipped with a two-photon laser (MaiTai BB, Spectra-Physics) and HCX-PL APO 100x/1.40-0.70 oil objective. Laurdan fluorescence was excited with a 770-nm wavelength light, and the emitted light was collected in the 400–460 nm range for one channel and the 470–530 nm range for the other channel. General polarization (GP) was calculated using the following formula, as previously described (27):

$$\text{GP }=\;\left(I_{400-460}-G\;{\times \;I}_{470-530}\right)/\left(I_{400-460}+G\times I_{470-530}\right),$$

where *I* is light intensity and G is a correction factor calculated from two variables: a known GP value for Laurdan in DMSO at 22°C, and the GP value of the Laurdan stock solution (500 µM) determined in each experiment. GP images (512 × 512 pixels) were analyzed and pseudocolored with ImageJ1.44n software (NIH Image). The GP values, which reflect the extent of water penetration into a lipid bilayer, were used as an indicator of the membrane lipid order.

### Imaging of Mitochondrial Membrane Lipid Order

The lipid order of mitochondrial membranes was examined using real-time imaging with the solvatochromic fluorescent probe NRMito (a kind gift from Dr. Andrew S. Klymchenko), a Nile Red derivative-bearing chemical groups for the specific targeting of mitochondria (22). HAECs were incubated with 20 nM NRMito for 45 min at 37°C. The localization of NRMito in the mitochondria of HAECs was detected using 50 nM of MitoTracker Green FM (Invitrogen). The NRMito imaging was performed using a confocal laser-scanning microscope (TCS SP2 AOBS, Leica Microsystems) with an HCX-PL APO 63x/1.40-0.60 oil objective and argon laser at 37°C. NRMito fluorescence was excited with light at a 514-nm wavelength, and the emitted light was collected in the 550–600 nm range for one channel and the 600–650 nm range for the other channel. The ratiometric images were analyzed using MetaMorph software, version 7.7 (Molecular Devices).

### Imaging of Mitochondrial ATP

The mitochondrial ATP was imaged using a FRET-based ATP biosensor targeted at the mitochondrial matrix (mitAT1.03; a kind gift from Dr. Hiromi Imamura), as previously described (28). The cDNA of mitAT1.03 was ligated and cloned into the *Sal*I/*Not*I sites of a pAd-CMV-V5-DEST Gateway Vector (Thermo Fisher Scientific), and the adenovirus vector containing mitAT1.03 cDNA (Ad-mitAT1.03) was constructed, as described previously (20). Cultured HAECs were infected with 50 pfu/cell of Ad-mitAT1.03. Three to five days after Ad-mitAT1.03 infection, the HAECs (maintained at 37°C) were imaged using an ECLIPSE Ti-E inverted microscope (Nikon) with ×100 Apo TIRF oil objective (NA = 1.49) using a water-cooling electron multiplier CCD camera (ImagEM C9100-13; Hamamatsu), controlled by HCImage software, version 4.3.5.6 (Hamamatsu). Dual-emission ratio imaging of mitAT1.03 was performed using an FF01-427/10 excitation filter (Semrock), an FF458-Di01 dichroic mirror, and a Dual View Multichannel Imaging System (DV2, Photometrics) equipped with two emission filters (FF01-483/32 for CFP and FF01-542/27 for YFP). The images were analyzed using MetaMorph software, version 7.7 (Molecular Devices).

### Imaging of Mitochondrial H_2_O_2_ Release

Mitochondrial reactive oxygen species (ROS) were imaged using a mitochondria matrix targeted H_2_O_2_ probe, HyPer7 (29). The cDNA of pCS2 + MLS-HyPer7 was purchased from Addgene (Plasmid ID: 136470) and was transfected in HAECs using the Neon Transfection System (Invitrogen), according to the manufacturer’s recommendations. Three days after transfection, real-time imaging was performed using an ECLIPSE Ti-E inverted microscope (Nikon) with ×40 Plan Apo silicon oil λS objective (1.25 numerical aperture [NA]) using a water-cooling electron multiplier CCD camera (ImagEM C9100-13; Hamamatsu), controlled by NIS Element software, version 4.30 (Nikon). The fluorescence of the HyPer7 probes was excited sequentially via FF02-438/24 and FF01-500/24 band-pass excitation filters (Semrock). Emissions were collected every 0.1 s using a FF01-542/27 band-pass emission filter cube equipped with a FF520-Di02-25x36 dichroic mirror (Semrock). Images of the HyPer7 fluorescence intensity ratio (F500/F438) were analyzed using MetaMorph software, version 7.7 (Molecular Devices).

### Statistical Analysis

The results were presented as the means ± SD. Statistical significance was evaluated using an ANOVA with the Bonferroni adjustment applied to the results of post hoc *t* tests. The statistical analysis was performed using SPSS software (SPSS Inc.). A *P* value of <0.01 was regarded as being statistically significant.

## RESULTS

### Laminar Flow Rapidly Decreased the Lipid Order of Plasma Membranes, Whereas Disturbed Flow Increased the Lipid Order

**Figure 2.**
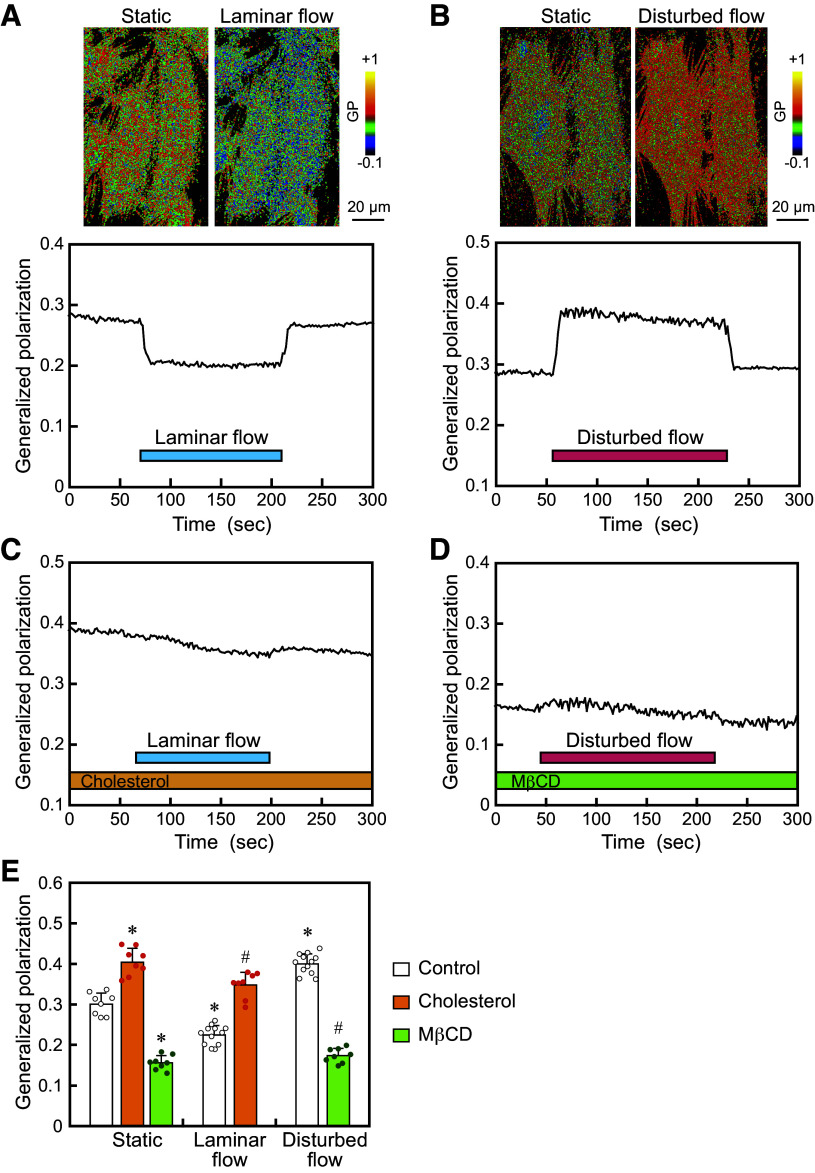
Laminar vs. disturbed flow effects on the lipid order of plasma membranes. *A, B*: 3 D-reconstructed Laurdan generalized polarization (GP) pseudocolor images of human aortic ECs (HAECs) before and 2 min after laminar flow or disturbed flow. The colors represent different GP values, an indicator of membrane lipid order, according to the scale. The pseudocolor images and temporal changes in GP values showed that the EC membrane lipid order clearly decreased in response to laminar flow, while it clearly increased in response to disturbed flow. *C*: addition of cholesterol (100 µM) to ECs prevented the laminar flow-induced decrease in plasma membrane lipid order. *D*: treatment of ECs with methyl-β-cyclodextrin (MβCD; 10 mM), a membrane cholesterol depleting agent, abolished the disturbed flow-induced increase in plasma membrane lipid order. *E*: quantitative analysis of the changes in the GP values induced by laminar flow and disturbed flow, with or without the addition of cholesterol and treatment with MβCD. Values are the means ± SD of data obtained from the samples shown as points. *P < 0.01 vs. static control. #*P* < 0.01 vs. each flow control.

HAECs were subjected to laminar flow with zero NtransWSS or disturbed flow with 0.2 or more NtransWSS (average, 0.4) in flow-loading devices (Fig. 1), and the resulting changes in the lipid order of their plasma membranes were examined using real-time Laurdan imaging. Fluorescent images of Laurdan-labeled HAECs were obtained, and images of GP were reconstructed as described in the Materials and Methods section. The GP images shown in pseudocolor demonstrated a heterogeneous distribution of GP values across the cell surface, indicating that EC membranes have a nonuniform lipid order in which both liquid-ordered states with high GP values and liquid-disordered states with low GP values coexist (Fig. 2, *A* and *B*).

When laminar flow was applied to the cells, the GP images showed a decrease in the lipid order over the entire plasma membrane (Fig. 2*A*). Temporal changes in lipid order were quantified by placing regions of interest (ROIs) throughout the cell. The GP values decreased immediately after the onset of the flow, plateaued thereafter, and returned to the control level after the flow ceased. In contrast, when the cells were subjected to disturbed flow, the GP images showed an increase in the lipid order over the entire plasma membrane (Fig. 2*B*). The GP values began to increase immediately after the application of the disturbed flow, remained at an increased level, and then returned to the control level after the flow ceased. The data obtained from many cells confirmed that EC membranes exhibit contrasting responses to laminar and disturbed flow by changing their membrane lipid order in opposite directions (Fig. 2*E*). This indicates that the EC plasma membranes distinguish between the two flow patterns through their lipid order responses.

The addition of cholesterol to the cells increased the lipid order of the plasma membranes under static conditions and abolished the lipid-order lowering effect of laminar flow (Fig. 2, *C* and *E*). On the other hand, the treatment of ECs with methyl-β-cyclodextrin (MβCD), which extracts membrane cholesterol, reduced the lipid order of plasma membranes under static conditions and markedly prevented the lipid-order increasing effect of disturbed flow (Fig. 2, *D* and *E*). These findings indicate that adding or removing cholesterol to cells directly alters the lipid order of plasma membranes and can prevent the responses of the membrane lipid order to laminar and disturbed flow.

### Laminar Flow Rapidly Decreased the Lipid Order of Mitochondrial Membranes, Whereas Disturbed Flow Increased the Lipid Order

**Figure 3.**
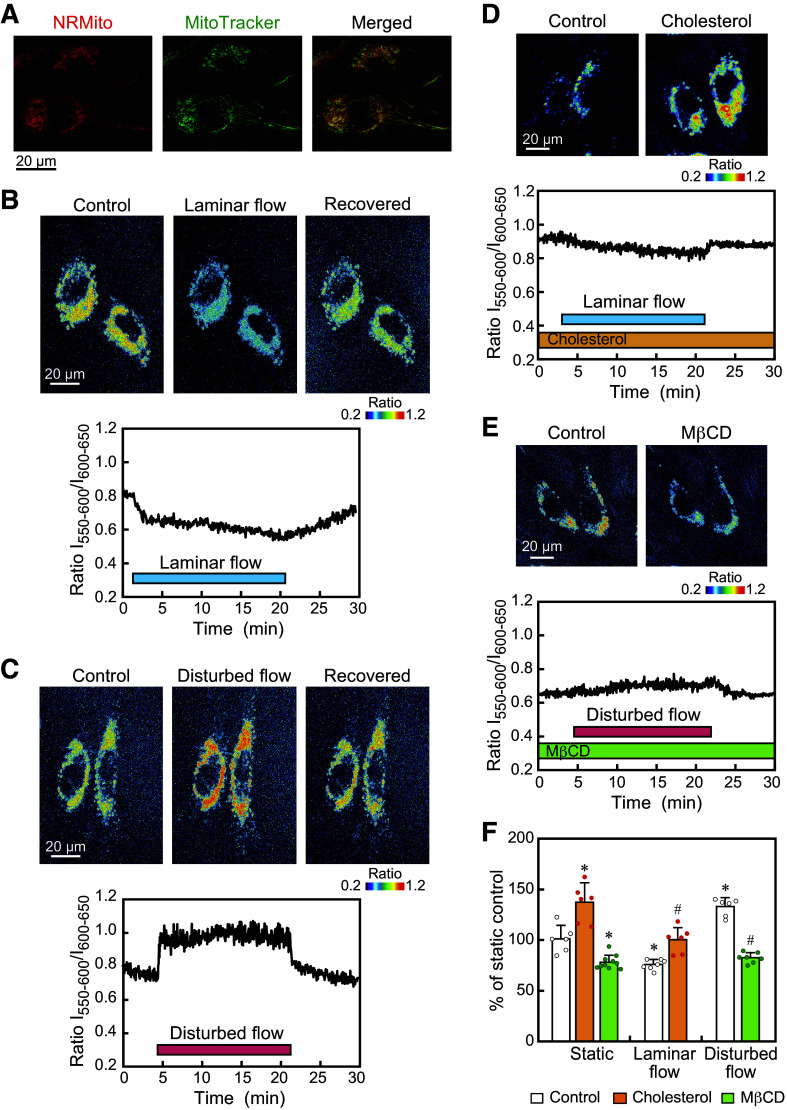
Laminar vs. disturbed flow effects on the lipid order of mitochondrial membranes. *A*: confocal images of live human aortic endothelial cells (HAECs) expressing the solvatochromic probe NRMito (in red) and labeled with the mitochondrion selective dye MitoTracker (in green). NRMito was correctly located in mitochondria. *B*: pseudocolor images of the NRMito fluorescence intensity ratio obtained before, during, and after the application of laminar flow. These images and the temporal change in the NRMito ratio showed that the mitochondrial membrane lipid order rapidly decreased in response to laminar flow. *C*: the pseudocolor images and the temporal change in the NRMito ratio showed that the lipid order of the mitochondrial membranes rapidly increased in response to disturbed flow. *D*: effects of cholesterol addition to ECs on the mitochondrial membrane lipid order and its response to laminar flow. The pseudocolor images obtained 6 h after the addition of cholesterol (100 µM) showed an increase in the lipid order of the mitochondrial membranes. The temporal change in the NRMito ratio showed that cholesterol addition prevented the laminar flow-induced decrease in the lipid order. *E*: effects of cholesterol extraction by treating ECs with MβCD (10 mM) on the mitochondrial membrane lipid order and its response to disturbed flow. The pseudocolor images obtained 30 min after MβCD treatment showed a decrease in the lipid order of the mitochondrial membrane. The temporal change in the NRMito ratio showed that cholesterol extraction abolished the disturbed flow-induced increase in the lipid order. *F*: quantitative analysis of the changes in the NRMito ratio induced by laminar flow and disturbed flow, with or without the addition of cholesterol and treatment with MβCD. Values are the means ± SD of the data obtained from samples shown as points. *P < 0.01 vs. control. #*P* < 0.01 vs. each flow control.

HAECs were exposed to laminar flow or disturbed flow, and changes in the lipid order of the mitochondrial membranes were examined using real-time imaging with the solvatochromic fluorescent probe NRMito (22). The localization of NRMito in HAECs coincided precisely with the mitochondria, as shown by live-cell imaging using the mitochondrion-selective dye MitoTracker (Fig. 3*A*). Ratiometric images of NRMito-labeled HAECs were reconstructed as described in materials and methods and displayed in pseudocolor.

Pseudocolor images showed that laminar flow reversibly decreased the NRMito ratio, i.e., the lipid order of the mitochondrial membrane (Fig. 3*B*). As shown in the temporal change in the NRMito ratio, the lipid order decreased right after the onset of laminar flow and plateaued thereafter; the decreased level returned to the control level after the cessation of flow. In contrast, pseudocolor images demonstrated that disturbed flow reversibly increased the lipid order of the mitochondrial membrane (Fig. 3*C*). The temporal change showed that the lipid order began to increase immediately after the application of disturbed flow, remained at an increased level, and then returned to the control level after the flow ceased. The data obtained from many cells confirmed that mitochondrial membranes exhibit contrasting responses to laminar and disturbed flow by changing their membrane lipid order in opposite directions (Fig. 3*F*).

The addition of cholesterol to cells increased the mitochondrial membrane lipid order of HAECs under static conditions and markedly suppressed the laminar flow-induced decrease in the lipid order (Fig. 3, *D* and *F*). In contrast, cholesterol extraction by treating the cells with MβCD significantly decreased the mitochondrial membrane lipid order of cells under static conditions, and it clearly prevented the disturbed flow-induced increase in the lipid order (Fig. 3, *E* and *F*). These findings indicate that adding or removing cholesterol to cells affects the lipid order of mitochondrial membranes and can prevent the effects of laminar and disturbed flow on mitochondrial membrane lipid order.

### Laminar Flow Rapidly Increased Mitochondrial ATP Production, Whereas Disturbed Flow Decreased ATP Production

**Figure 4.**
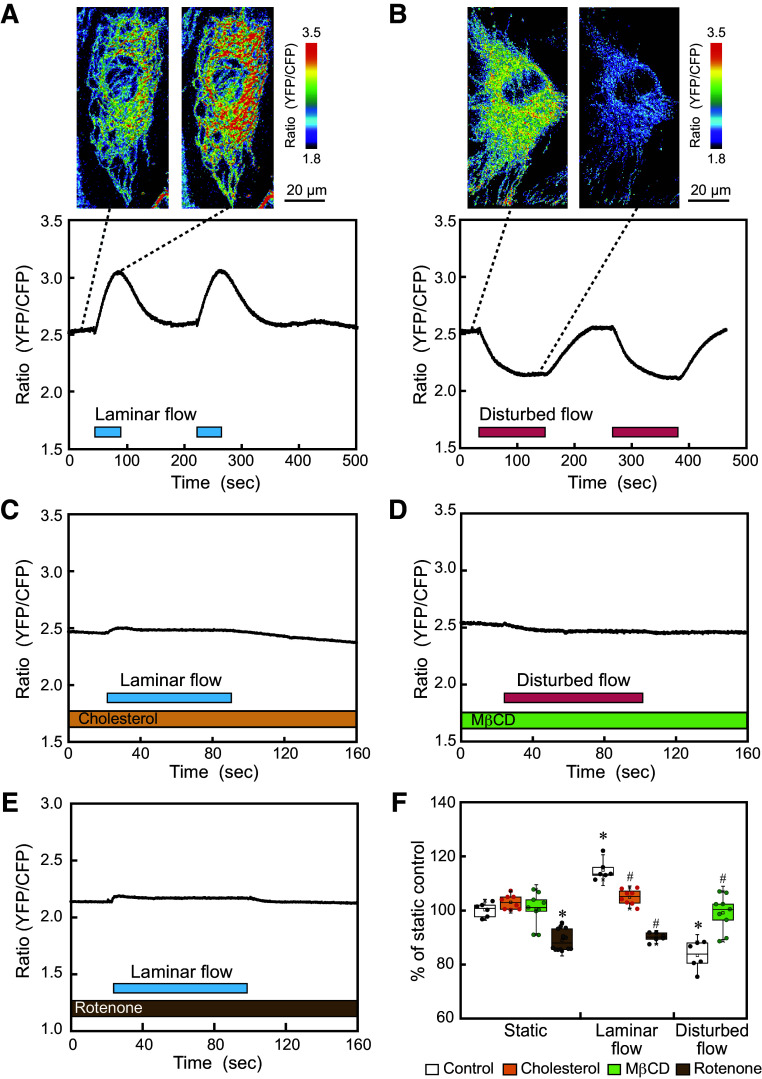
Laminar vs. disturbed flow effects on mitochondrial adenosine triphosphate (ATP) production. Human aortic endothelial cells (HAECs) were exposed to laminar flow or disturbed flow and changes in mitochondrial ATP production were examined using real-time imaging with the mitochondria-targeting ATP biosensor mitAT1.03. *A*: mitochondrial ATP responses to laminar flow. Pseudocolor images of the mitAT1.03 fluorescence ratio (YFP/CFP) exhibited an increase in ATP levels when the cell was exposed to laminar flow. The temporal changes in the mitAT 1.03 ratio showed that two similar responses of increasing ATP levels occurred for two repeated laminar flow loadings. *B*: mitochondrial ATP responses to disturbed flow. Pseudocolor images exhibited a decrease in ATP levels when the cell was exposed to disturbed flow. Temporal changes showed that two similar responses of decreasing ATP levels occurred for two repeated disturbed flow loadings. *C*: addition of cholesterol (100 µM) to ECs markedly inhibited the laminar flow-induced increase in mitochondrial ATP production. *D*: cholesterol extraction by treating ECs with MβCD (10 mM) abolished the disturbed flow-induced decrease in mitochondrial ATP production. *E*: treatment of ECs with rotenone (5 µM), an inhibitor of the mitochondrial electron transport system, lowered the mitochondrial ATP levels in cells under static conditions and abolished the ATP increasing effect of laminar flow. *F*: quantitative analysis of the changes in mitochondrial ATP production induced by laminar flow and disturbed flow, with or without cholesterol, MβCD, or rotenone. Values are the means ± SD of the data obtained from samples shown as points. *P < 0.01 vs. control. #*P* < 0.01 vs. each flow control.

HAECs were exposed to laminar flow or disturbed flow, and changes in the mitochondrial ATP levels were examined using real-time imaging with mitAT1.03 (28). Pseudocolor images of the YFP/CFP ratio showed increased ATP levels over the entire mitochondria when the cell was exposed to laminar flow (Fig. 4, *A* and *F*). In contrast, cells subjected to disturbed flow exhibited decreased ATP levels (Fig. 4, *B* and *F*). Temporal changes in ATP levels were quantified by defining ROIs throughout the cell. The ATP level quickly increased in response to laminar flow but decreased in response to disturbed flow, and both the increased and decreased ATP levels returned to their original levels after flow ceased. Similar reactions occurred in response to a second flow loading, indicating that the mitochondrial responses to laminar and disturbed flow were reversible.

The addition of cholesterol to the cells abolished the laminar flow-induced increase in mitochondrial ATP levels (Fig. 4, *C* and *F*), and treatment of cells with MβCD prevented the disturbed flow-induced decrease in mitochondrial ATP levels (Fig. 4, *D* and *F*). These findings indicate that changes in membrane physical properties, such as lipid order and cholesterol content, are closely linked to the effects of laminar and turbulent flow on mitochondrial ATP production.

Treatment of cells with rotenone, an inhibitor of mitochondrial electron transport chain complex I, abolished the laminar-flow-induced increase in ATP levels, indicating that the changes in ATP levels were due to changes in mitochondrial oxidative phosphorylation (Fig. 4, *E* and *F*).

### Laminar Flow Decreased Mitochondrial H_2_O_2_ Release, Whereas Disturbed Flow Increased H_2_O_2_ Release

**Figure 5.**
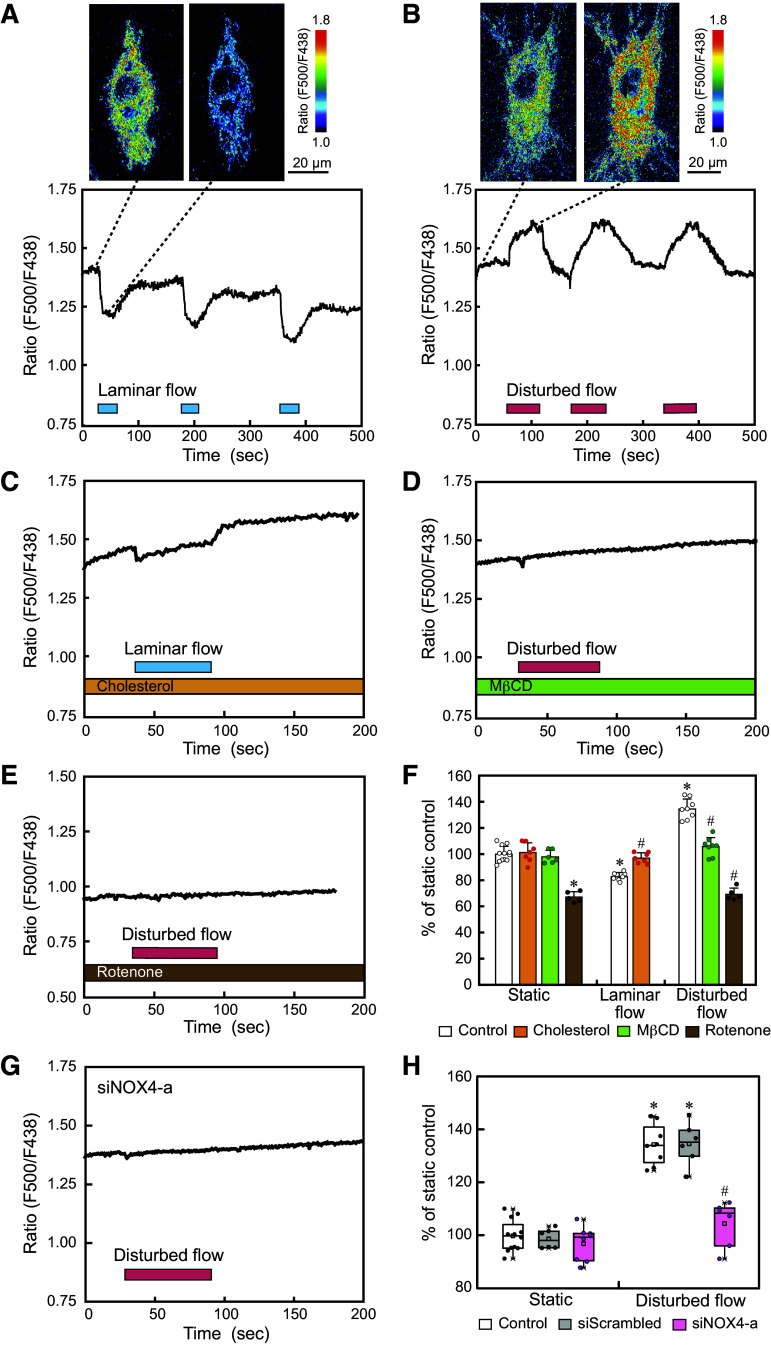
Laminar vs. disturbed flow effects on mitochondrial H_2_O_2_ release. Human aortic endothelial cells (HAECs) were exposed to laminar flow or disturbed flow, and changes in mitochondrial H_2_O_2_ release were examined using real-time imaging with a mitochondrial matrix-targeted H_2_O_2_ sensor probe, HyPer7. *A*: mitochondrial H_2_O_2_ responses to laminar flow. Pseudocolor images of the HyPer7 fluorescence intensity ratio (F500/F438) exhibited a decrease in H_2_O_2_ levels when the cell was exposed to laminar flow. The temporal changes in the HyPer7 ratio showed that three similar responses of decreasing H_2_O_2_ levels occurred for three repeated laminar flow loadings. *B*: mitochondrial H_2_O_2_ responses to disturbed flow. Pseudocolor images exhibited an increase in H_2_O_2_ levels when the cell was exposed to disturbed flow. The temporal changes showed that three similar responses of increasing H_2_O_2_ levels occurred for three repeated disturbed flow loadings. *C*: addition of cholesterol (100 µM) to ECs markedly inhibited the laminar flow-induced decrease in mitochondrial H_2_O_2_ release. *D*: Cholesterol extraction by treating ECs with MβCD (10 mM) abolished the disturbed flow-induced increase in mitochondrial H_2_O_2_ release. *E*: treatment of ECs with rotenone (5 µM) abolished the disturbed-flow-induced H_2_O_2_ increase. *F*: quantitative analysis of the changes in mitochondrial H_2_O_2_ release induced by laminar flow and disturbed flow, with or without cholesterol, MβCD, or rotenone. Values are the means ± SD of the data obtained from the samples shown as points. *P < 0.01 vs. control. #*P* < 0.01 vs. each flow control. *G*: knockdown of Nox4 expression with siNOX4-a blocked the disturbed flow-induced H_2_O_2_ increase. *H*: quantitative analysis of the changes in mitochondrial H_2_O_2_ release induced by disturbed flow, with or without siScrambled or siNOX4-a. Values are the means ± SD of the data obtained from the samples shown as pointes. *P < 0.01 vs. control. #*P* < 0.01 vs. each flow control.

HAECs were exposed to laminar flow or disturbed flow, and changes in mitochondrial H_2_O_2_ release were examined using real-time imaging with HyPer7 (29). Pseudocolor images of the HyPer7 fluorescence intensity ratio (F500/F438) exhibited a clear decrease in H_2_O_2_ levels when the cells were exposed to laminar flow (Fig. 5*A*). Temporal changes in the H_2_O_2_ level were quantified by setting ROIs throughout the cell, and three similar reductions in H_2_O_2_ levels were observed for three repeated laminar flow loadings. In contrast, pseudocolor images exhibited a clear increase in H_2_O_2_ levels when the cells were exposed to disturbed flow (Fig. 5*B*). Temporal changes showed three similar increases in H_2_O_2_ levels in response to three repeated disturbed flow loadings. Both the laminar flow-induced decrease and disturbed flow-induced increase in H_2_O_2_ levels returned to the control level after each flow ceased, indicating that the mitochondrial H_2_O_2_-releasing responses were reversible. Quantitative analysis of many cells confirmed that mitochondrial H_2_O_2_ release exhibited contrasting responses, decreasing with laminar flow and increasing with disturbed flow (Fig. 5*F*).

The addition of cholesterol to ECs markedly suppressed the laminar flow-induced decrease in the mitochondrial H_2_O_2_ release (Fig. 5, *C* and *F*). In contrast, cholesterol extraction by treating ECs with MβCD abolished the disturbed flow-induced increase in mitochondrial H_2_O_2_ release (Fig. 5, *D* and *F*). These findings indicate that changes in membrane physical properties, such as lipid order and cholesterol content, are closely linked to the effects of laminar and disturbed flows on mitochondrial H_2_O_2_ release.

Furthermore, when ECs were treated with rotenone, disturbed flow did not increase H_2_O_2_ levels in ECs, indicating that the mitochondrial electron transfer system was involved in the disturbed flow-induced increase in H_2_O_2_ levels (Fig. 5, *E* and *F*).

**Figure 6.**
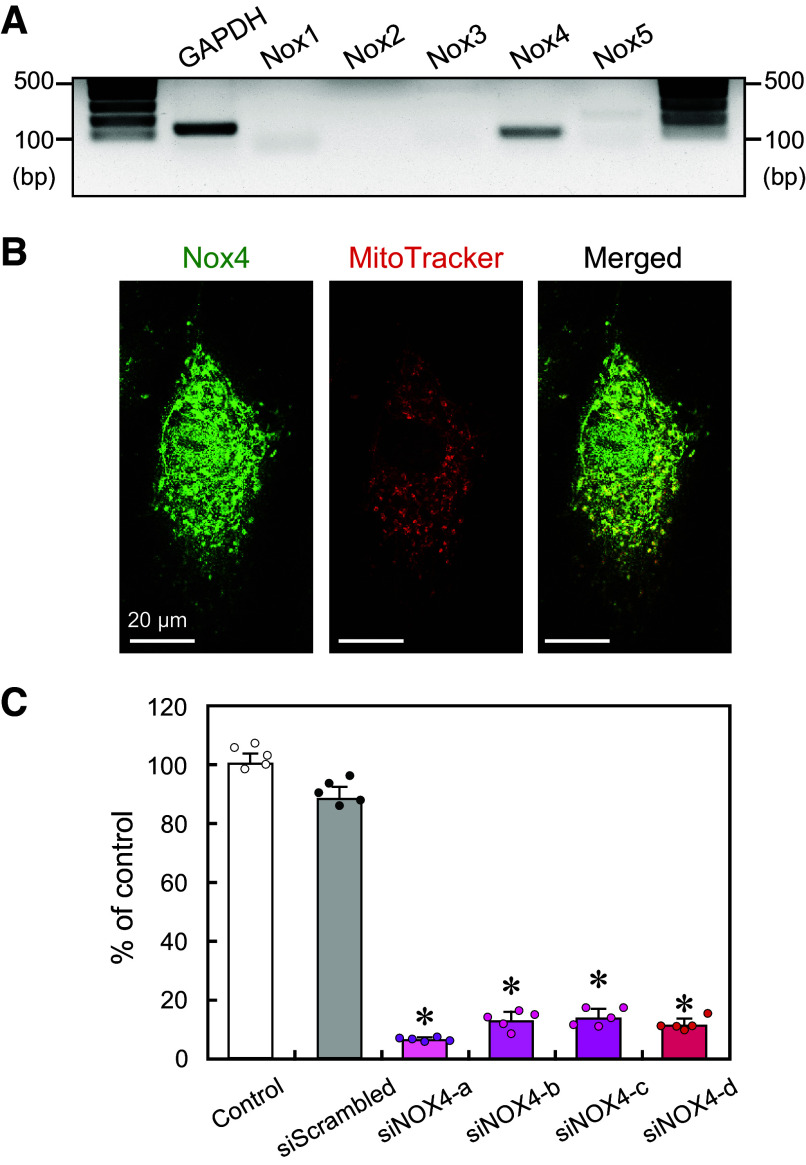
*A*: the mRNA expression of Nox isoforms was determined using RT-PCR. Nox4 was predominantly expressed, but Nox1, 2, 3, and 5 were barely expressed in human aortic endothelial cells (HAECs). *B*: confocal microscopic images of HAECs were stained with anti-Nox4 antibody (in green) and labeled with the mitochondrion selective dye MitoTracker Deep Red (in red). The majority of Nox4 was localized to the mitochondria. *C*: quantitative mRNA expression of Nox4 determined by RT-qPCR. All four siRNAs (siNOX4-a, b, c, d) effectively suppressed the mRNA expression of Nox4. Values are the means ± SD of the data obtained from samples shown as points. *P < 0.01 vs. control.

Next, we examined whether NADPH oxidase (Nox) was involved in the increase in H_2_O_2_ levels. Real-time PCR showed that the Nox isoform 4 (Nox4) was predominantly expressed at the mRNA level and that other isoforms, including Nox1, 2, 3, and 5, were barely expressed in the HAECs used in this study (Fig. 6*A*). Immunostaining with a Nox4 antibody showed that the Nox4 protein was predominantly localized to mitochondria in HAECs (Fig. 6*B*). Small-interfering RNA (siRNA), siNOX4-a, effectively knocked down the gene expression of Nox4 in HAECs, thereby abolishing the disturbed flow-induced increase in H_2_O_2_ levels (Fig. 6*C* and Fig. 5, *G* and *H*). Taken together, these findings indicate that the increased H_2_O_2_ originated from ROS produced through the cooperation of the mitochondrial electron transport system and Nox4.

## DISCUSSION

A variety of in vitro models have been used to study the effect of disturbed flow on ECs (3). These include disturbed flow-loading systems using parallel-plate flow chambers or cone-and-plate viscometers. Among these models, the flow chamber system has the advantage of providing a simple, predictable, and controllable disturbed flow to the cells. In this study, we used a flow system in which a step is placed upstream of the flow path of the parallel-plate flow chamber to generate disturbed flow. This system allows direct microscopic observation of the cells under flow conditions and real-time imaging with various photoaffinity probes. More importantly, the system was able to reproduce the desired in vivo disturbed flow by adjusting the height of the step based on CFD analysis of the medium flow in the chamber. As a hemodynamic metric that quantitatively indicates the multidirectional character of disturbed flow, we used the transverse wall shear stress (transWSS), which is calculated as the time-average of wall shear stress components perpendicular to the mean flow direction. TransWSS was shown to be an important factor in determining the site of origin of diet-induced plaques at intercostal artery bifurcation orifices in rabbits (30), and the NtransWSS, which corresponds to transWSS divided by the time-averaged WSS, was correlated most strongly with the location and size of human cerebral aneurysms among various hemodynamic metrics, including the time-averaged WSS gradient, oscillatory shear index, and gradient oscillatory number (23).

The present study showed that the plasma membrane immediately responds to disturbed flow by increasing lipid order. This was the opposite of the response to laminar flow, in which the lipid order was reduced, indicating that the plasma membrane itself can differentiate flow patterns between laminar and disturbed flows. Our previous study showed that a similar decrease in lipid order occurred when the artificial membranes of giant unilamellar vesicles were exposed to laminar flow, which indicates that the lipid order response is a physical phenomenon that does not involve participation by any membrane proteins, the cytoskeleton, or biological activities of living cells (11). Changes in the physical properties of the plasma membrane, such as lipid order, fluidity, and viscosity, are known to have a direct effect on the conformation and dynamics of lipid and protein molecules present in the plasma membrane, modifying their function (31, 32). Numerous studies have shown that flow stimuli to ECs activate a variety of membrane molecules, including ion channels such as Piezo-1 and TRPV4, receptors such as G protein coupling receptors and vascular endothelial growth factor receptors (VEGFRs), and adhesion molecules such as vascular endothelium cadherin and platelet-EC adhesion molecule-1 (9, 33–37). Accordingly, it is highly possible that changes in the lipid order of plasma membranes are involved in the flow-induced activation of many, if not all, of the above-mentioned membrane molecules. Taken together, ECs seem to have a flow-sensing and signaling mechanism in which their plasma membranes act as a sensor to separate laminar and disturbed flow, and membrane-associated molecules, microdomains, and subcellular organelles act as transducers to transmit information downstream (6).

The current study demonstrated that flow stimuli to ECs changed the lipid order of not only plasma membranes but also mitochondrial membranes. Similar to the effects on plasma membranes, laminar flow decreased the lipid order of the mitochondrial membranes, while disturbed flow increased the lipid order. How such responses occur in mitochondrial membranes that are not directly exposed to flow remains unknown. Several possible mechanisms can be postulated. Flow-induced changes in the lipid composition of the plasma membrane, such as changes in cholesterol content, may lead to similar changes in the mitochondrial membrane. In fact, cell treatments involving the addition or removal of cholesterol blocked the laminar and disturbed flow-induced changes in lipid order in both plasma and mitochondrial membranes. Another mechanism is that since mitochondria are bound to the cytoskeleton (for example, to actin filaments), mechanical stress transmitted through the cytoskeleton may act on mitochondrial membranes to alter their lipid order (38, 39). Alternatively, when fluid shear stress was applied to liposomes that were made of artificial lipid bilayers, the flow was reportedly generated within the liposomes (40, 41). Although the mechanism responsible for this phenomenon is unclear, shear stress may induce the flow of liquid components within ECs, directly affecting mitochondrial membranes. Understanding this process will require further research.

In ECs, the production of ATP as energy is mainly dependent on the glycolytic system, and mitochondria-produced ATP is thought to act as a signaling molecule (15). Our previous study showed that mitochondria play a critical role in EC signaling of laminar flow by increasing ATP production, leading to purinergic Ca^2+^ signaling (20); the present study revealed that mitochondria are also involved in the signaling of disturbed flow. Real-time imaging with Hyper7 demonstrated that disturbed flow caused an immediate increase in mitochondrial H_2_O_2_ release. This response was abolished by the treatment of cells with rotenone, an electron transfer system inhibitor. Furthermore, Nox4, which catalyzes the production of a superoxide free radical, was shown to be involved in the disturbed flow-induced H_2_O_2_ release. Thus, the increased H_2_O_2_ was thought to originate from ROS produced by the cooperation of the mitochondrial electron transport system and Nox4. Thus, EC mitochondria appeared to transmit information regarding disturbed flow downstream via ROS signaling.

In the current study, we found that changes in the physical properties of plasma and mitochondrial membranes were closely linked to changes in mitochondrial functions, such as ATP and ROS production. The blockage of flow-induced changes in the lipid order of both membranes by adding or removing cholesterol abolished the laminar flow- or disturbed flow-induced ATP production or H_2_O_2_ release, respectively. Concerning how changes in the lipid order of mitochondrial membranes affect oxidative phosphorylation to produce ATP or ROS, several possible mechanisms were proposed: 1) changing the permeability of the membrane to oxygen and other substrates necessary for respiration, 2) affecting the function of enzymes working in electron transport systems, and 3) influencing the assembly and function of respiratory chain complexes by altering the diffusion of molecules within the membrane (21).

To date, many studies have shown that oscillatory flow, a pattern of disturbed flow, increases ROS production in ECs (42–46). When cultured ECs, such as human umbilical ECs, murine aortic ECs, and bovine aortic ECs, were exposed to oscillatory flow with a velocity that oscillated back and forth with a periodicity of ±3–5 dyne/cm^2^, 1 Hz, the production of superoxide (O_2_^−^) and H_2_O_2_ significantly increased from 30 min to 24 h later. NADPH oxidases, including Nox1, Nox2, and Nox4, were also shown to be involved in oscillatory-flow-induced ROS production, although the isoform of Nox involved differed depending on the cell type. The present study showed that the expression of Nox4 was prominent in HAECs, but Nox1, Nox2, and Nox5 were barely expressed and that Nox4-mediated ROS production contributed considerably to the disturbed flow-induced increase in mitochondrial H_2_O_2_ release. Recently, Nox4 was shown to be localized to the mitochondrial inner membrane in human renal epithelial cells and to have an ATP-binding motif; its superoxide generation activity was also found to be negatively regulated by mitochondrial ATP levels (47). Thus, this study showed that disturbed flow had the effect of lowering the mitochondrial ATP levels, which may have activated Nox4 to increase ROS production within the mitochondrial compartment.

Mitochondria-produced ROS were once thought to be harmful byproducts that impaired both mitochondria and cellular functions. Recently, however, mitochondrial ROS have been shown to be deeply involved in the maintenance of cellular homeostasis and adaptations to environmental stresses (48–50). Among ROS, H_2_O_2_ is highly stable, has a long half-life in vivo, and can pass freely through cell membranes, as it has no electrical charge. When released from ECs, H_2_O_2_ acts as an endothelial hyperpolarizing factor in smooth muscles and dilates blood vessels (51). H_2_O_2_ also plays a role in angiogenesis and vascular remodeling by augmenting EC migration and proliferation via the phosphorylation of VEGFRs (52, 53). In addition, H_2_O_2_ mediates the expression of heme oxygenase-1 (54), which protects cells from inflammation, and the stabilization of hypoxia-inducible factor-1, which is required for the adaptive response to hypoxia. ROS also cause reversible post-translational modifications of proteins via oxidation and regulate several signaling pathways (55). Therefore, H_2_O_2_ released by mitochondria is thought to act as a second messenger to inform cells of disturbed flow and to trigger cellular responses that allow adaptation via redox signaling. However, the specific molecular targets of mitochondrial H_2_O_2_ produced in response to disturbed flow are currently unknown, and identifying these targets will be an important topic for future research. On the other hand, as a long-term effect of disturbed flow, if ECs are exposed to cardiovascular risk factors such as hypertension, dyslipidemia, and hyperglycemia, and if mitochondria cooperate with other intracellular sources of ROS to produce excessive ROS, EC dysfunction and the activation of proinflammatory pathways would occur; if sustained, this would lead to the development of atherosclerotic plaques and aneurysms (49).

**Figure 7.**
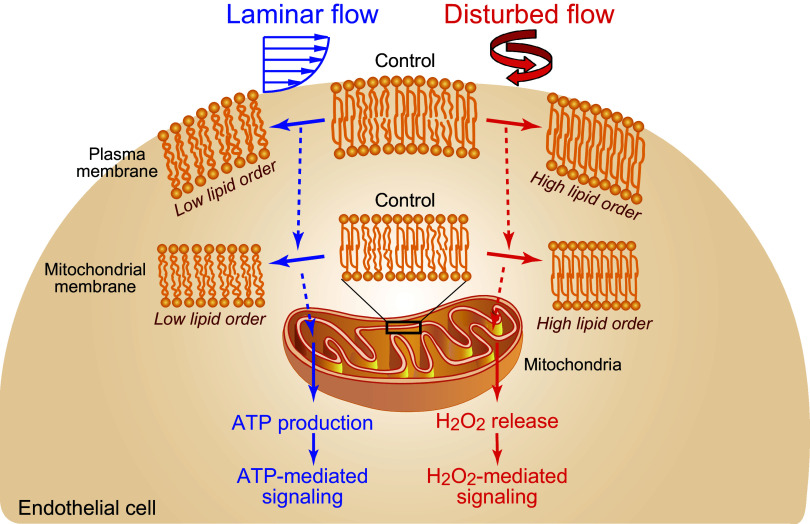
Schematic diagram of the proposed sensing of laminar and disturbed flows and subsequent signaling in endothelial cells (ECs). ECs sense laminar and disturbed flows through changes in the lipid order of their plasma membranes, with laminar flow decreasing, and disturbed flow increasing the lipid order. Similar changes in the lipid order also occur almost simultaneously in the mitochondrial membranes. Changes in membrane lipid order are linked to changes in the mitochondrial functions (i.e., increased ATP production for laminar flow and increased H_2_O_2_ release for disturbed flow). Thus, ECs conduct distinct sensing of these two flow patterns and activate appropriate signalings via changes in the lipid order of the plasma and mitochondrial membranes. It remains unknown how the different flow patterns change the lipid order of the mitochondrial membranes, which are not directly exposed to the flows, and how changes in the membrane lipid order alter the mitochondrial functions. ATP, adenosine triphosphate; H_2_O_2_, hydrogen peroxide; ROS, reactive oxygen species.

In summary, the current study demonstrated that ECs exhibit distinct sensing of laminar and disturbed flows and activate the appropriate signalings and that both the plasma and mitochondrial membranes of the ECs play critical roles in these processes, that is, they mediate the intracellular signaling specific to each flow pattern through altering their lipid order in opposite directions. We found that the changes in the membrane lipid order were closely linked to changes in the mitochondrial functions, i.e., increased ATP production for laminar flow and increased H_2_O_2_ release for disturbed flow. Thus, laminar flow information seems to be transmitted downstream as ATP-mediated purinergic signaling, whereas disturbed flow information is transmitted as ROS-mediated redox signaling (Fig. 7). However, a number of issues still remain to be clarified, such as the biophysical or thermodynamic mechanisms by which shear stress alters the plasma membrane lipid order, the mechanism by which shear stress acting on the plasma membrane affects the mitochondrial membrane properties, and the mechanism by which changes in the mitochondrial membrane lipid order modulate the electron transfer system and Nox to produce ATP and ROS. In addition, it remains unclear if the differing lipid orders of the plasma and mitochondrial membranes observed in the cultured ECs are also manifested in the straight sections versus curved or bifurcated sections of arteries, where naturally occurring laminar and disturbed flows, respectively, prevail. Resolving these problems in the future will contribute not only to a better understanding of EC sensing and signaling mechanisms for different blood flow patterns, but also to the elucidation of their roles in vascular homeostasis and in pathological conditions, such as hypertension, thrombosis, aneurysms, and atherosclerosis. It is also expected to contribute to the development of new preventive and therapeutic strategies for these vascular diseases.

## DATA AVAILABILITY

Data will be made available upon reasonable request.

## GRANTS

This work was supported by Scientific Research from the Japan Agency for Medical Research and Development (AMED) CREST JP20gm0810006h (to K.Y.), as well as JSPS KAKENHI Grant Number JP21H03791 (to K.Y. and J.A.) and JKA (to K.Y.).

## DISCLOSURES

No conflicts of interest, financial or otherwise, are declared by the authors.

## AUTHOR CONTRIBUTIONS

K.Y. and J.A. conceived and designed research; K.Y., Y.S., and J.A. performed experiments; K.Y., Y.S., R.M., K.K., and J.A. analyzed data; K.Y., Y.S., and J.A. interpreted results of experiments; K.Y. and J.A. prepared figures; K.Y. and J.A. drafted manuscript; K.Y. and J.A. edited and revised manuscript; K.Y. and J.A. approved final version of manuscript.

## References

[1] Davies PF. Flow-mediated endothelial mechanotransduction. Physiol Rev 75: 519–560, 1995. doi:10.1152/physrev.1995.75.3.519. 7624393 PMC3053532

[2] Aoki T, Nishimura M, Matsuoka T, Yamamoto K, Furuyashiki T, Kataoka H, Kitaoka S, Ishibashi R, Ishibazawa A, Miyamoto S, Morishita R, Ando J, Hashimoto N, Nozaki K, Narumiya S. PGE_2_ - EP2 signalling in endothelium is activated by haemodynamic stress and induces cerebral aneurysm through an amplifying loop via NF-κB. Br J Pharmacol 163: 1237–1249, 2011. doi:10.1111/j.1476-5381.2011.01358.x. 21426319 PMC3144537

[3] Chiu JJ, Chien S. Effects of disturbed flow on vascular endothelium: pathophysiological basis and clinical perspectives. Physiol Rev 91: 327–387, 2011. doi:10.1152/physrev.00047.2009. 21248169 PMC3844671

[4] Simmons RD, Kumar S, Jo H. The role of endothelial mechanosensitive genes in atherosclerosis and omics approaches. Arch Biochem Biophys 591: 111–131, 2016. doi:10.1016/j.abb.2015.11.005. 26686737 PMC4747676

[5] Fang Y, Wu D, Birukov KG. Mechanosensing and mechanoregulation of endothelial cell functions. Compr Physiol 9: 873–904, 2019. doi:10.1002/cphy.c180020. 30873580 PMC6697421

[6] Yamamoto K, Ando J. Emerging role of plasma membranes in vascular endothelial mechanosensing. Circ J 82: 2691–2698, 2018. doi:10.1253/circj.CJ-18-0052. 30282847

[7] Butler PJ, Norwich G, Weinbaum S, Chien S. Shear stress induces a time- and position-dependent increase in endothelial cell membrane fluidity. Am J Physiol Cell Physiol 280: C962–C969, 2001. doi:10.1152/ajpcell.2001.280.4.C962. 11245613

[8] Butler PJ, Tsou TC, Li JY, Usami S, Chien S. Rate sensitivity of shear-induced changes in the lateral diffusion of endothelial cell membrane lipids: a role for membrane perturbation in shear-induced MAPK activation. FASEB J 16: 216–218, 2002. doi:10.1096/fj.01-0434fje. 11744620

[9] Gudi SR, Clark CB, Frangos JA. Fluid flow rapidly activates G proteins in human endothelial cells. Involvement of G proteins in mechanochemical signal transduction. Circ Res 79: 834–839, 1996. doi:10.1161/01.res.79.4.834. 8831508

[10] Haidekker MA, L'Heureux N, Frangos JA. Fluid shear stress increases membrane fluidity in endothelial cells: a study with DCVJ fluorescence. Am J Physiol Heart Circ Physiol 278: H1401–H1406, 2000. doi:10.1152/ajpheart.2000.278.4.H1401. 10749738

[11] Yamamoto K, Ando J. Endothelial cell and model membranes respond to shear stress by rapidly decreasing the order of their lipid phases. J Cell Sci 126: 1227–1234, 2013. doi:10.1242/jcs.119628. 23378020

[12] Yamamoto K, Furuya K, Nakamura M, Kobatake E, Sokabe M, Ando J. Visualization of flow-induced ATP release and triggering of Ca^2+^ waves at caveolae in vascular endothelial cells. J Cell Sci 124: 3477–3483, 2011. doi:10.1242/jcs.087221. 22010198

[13] Yamamoto K, Nogimori Y, Imamura H, Ando J. Shear stress activates mitochondrial oxidative phosphorylation by reducing plasma membrane cholesterol in vascular endothelial cells. Proc Natl Acad Sci USA 117: 33660–33667, 2020. doi:10.1073/pnas.2014029117. 33318210 PMC7776821

[14] Yamamoto K, Sokabe T, Matsumoto T, Yoshimura K, Shibata M, Ohura N, Fukuda T, Sato T, Sekine K, Kato S, Isshiki M, Fujita T, Kobayashi M, Kawamura K, Masuda H, Kamiya A, Ando J. Impaired flow-dependent control of vascular tone and remodeling in P2X4-deficient mice. Nat Med 12: 133–137, 2006. doi:10.1038/nm1338. 16327800

[15] Darley-Usmar V. The powerhouse takes control of the cell; the role of mitochondria in signal transduction. Free Radic Biol Med 37: 753–754, 2004. doi:10.1016/j.freeradbiomed.2004.05.026. 15304251

[16] Kluge MA, Fetterman JL, Vita JA. Mitochondria and endothelial function. Circ Res 112: 1171–1188, 2013. doi:10.1161/CIRCRESAHA.111.300233. 23580773 PMC3700369

[17] Quintero M, Colombo SL, Godfrey A, Moncada S. Mitochondria as signaling organelles in the vascular endothelium. Proc Natl Acad Sci USA 103: 5379–5384, 2006. doi:10.1073/pnas.0601026103. 16565215 PMC1459363

[18] Al-Mehdi A-B. Mechanotransduction of shear-stress at the mitochondria. In: Mitochondria. Advances in Biochemistry in Health and Disease, edited by Schaffer SW, Suleiman M-S. New York, NY: Springer, 2007, vol. 2, p. 169–181.

[19] Scheitlin CG, Julian JA, Shanmughapriya S, Madesh M, Tsoukias NM, Alevriadou BR. Endothelial mitochondria regulate the intracellular Ca^2+^ response to fluid shear stress. Am J Physiol Cell Physiol 310: C479–C490, 2016. doi:10.1152/ajpcell.00171.2015. 26739489 PMC4796279

[20] Yamamoto K, Imamura H, Ando J. Shear stress augments mitochondrial ATP generation that triggers ATP release and Ca^2+^ signaling in vascular endothelial cells. Am J Physiol Heart Circ Physiol 315: H1477–H1485, 2018. doi:10.1152/ajpheart.00204.2018. 30141983 PMC6297820

[21] Budin I, de Rond T, Chen Y, Chan LJG, Petzold CJ, Keasling JD. Viscous control of cellular respiration by membrane lipid composition. Science 362: 1186–1189, 2018. doi:10.1126/science.aat7925. 30361388

[22] Danylchuk DI, Jouard PH, Klymchenko AS. Targeted solvatochromic fluorescent probes for imaging lipid order in organelles under oxidative and mechanical stress. J Am Chem Soc 143: 912–924, 2021. doi:10.1021/jacs.0c10972. 33417447

[23] Fukuda S, Shimogonya Y, Yonemoto N, Group CAS; CFD ABO Study Group. Differences in cerebral aneurysm rupture rate according to arterial anatomies depend on the hemodynamic environment. AJNR Am J Neuroradiol 40: 834–839, 2019. doi:10.3174/ajnr.A6030. 30975650 PMC7053905

[24] Fukuda S, Shimogonya Y, Yonemoto N, Fukuda M, Watanabe A, Fujiwara K, Enomoto R, Hasegawa K, Yasoda A, Tsukahara T; NHO Carotid CFD Study Group. Hemodynamic risk factors for the development of carotid stenosis in patients with unilateral carotid stenosis. World Neurosurg 160: e353–e371, 2022. doi:10.1016/j.wneu.2022.01.019. 35026460

[25] Kamiya A, Bukhari R, Togawa T. Adaptive regulation of wall shear stress optimizing vascular tree function. Bull Math Biol 46: 127–137, 1984. doi:10.1007/BF02463726. 6713148

[26] Ito Y, Shih AM, Soni BK, Nakahashi K. Multiple marching direction approach to generate high-quality hybrid meshes. AIAA J 45: 162–167, 2007. doi:10.2514/1.23260.

[27] Shentu TP, Titushkin I, Singh DK, Gooch KJ, Subbaiah PV, Cho M, Levitan I. oxLDL-induced decrease in lipid order of membrane domains is inversely correlated with endothelial stiffness and network formation. Am J Physiol Cell Physiol 299: C218–C229, 2010. doi:10.1152/ajpcell.00383.2009. 20410437 PMC2928623

[28] Imamura H, Nhat KP, Togawa H, Saito K, Iino R, Kato-Yamada Y, Nagai T, Noji H. Visualization of ATP levels inside single living cells with fluorescence resonance energy transfer-based genetically encoded indicators. Proc Natl Acad Sci USA 106: 15651–15656, 2009. doi:10.1073/pnas.0904764106. 19720993 PMC2735558

[29] Pak VV, Ezeriņa D, Lyublinskaya OG, Pedre B, Tyurin-Kuzmin PA, Mishina NM, Thauvin M, Young D, Wahni K, Martínez Gache SA, Demidovich AD, Ermakova YG, Maslova YD, Shokhina AG, Eroglu E, Bilan DS, Bogeski I, Michel T, Vriz S, Messens J, Belousov VV. Ultrasensitive genetically encoded indicator for hydrogen peroxide identifies roles for the oxidant in cell migration and mitochondrial function. Cell Metab 31: 642–653.e6, 2020. doi:10.1016/j.cmet.2020.02.003. 32130885 PMC7088435

[30] Peiffer V, Sherwin SJ, Weinberg PD. Computation in the rabbit aorta of a new metric - the transverse wall shear stress - to quantify the multidirectional character of disturbed blood flow. J Biomech 46: 2651–2658, 2013. doi:10.1016/j.jbiomech.2013.08.003. 24044966 PMC3807647

[31] Levitan I, Fang Y, Rosenhouse-Dantsker A, Romanenko V. Cholesterol and ion channels. Subcell Biochem 51: 509–549, 2010. doi:10.1007/978-90-481-8622-8_19. 20213557 PMC2895485

[32] Phillips R, Ursell T, Wiggins P, Sens P. Emerging roles for lipids in shaping membrane-protein function. Nature 459: 379–385, 2009. doi:10.1038/nature08147. 19458714 PMC3169427

[33] Jin ZG, Ueba H, Tanimoto T, Lungu AO, Frame MD, Berk BC. Ligand-independent activation of vascular endothelial growth factor receptor 2 by fluid shear stress regulates activation of endothelial nitric oxide synthase. Circ Res 93: 354–363, 2003. doi:10.1161/01.RES.0000089257.94002.96. 12893742

[34] Li J, Hou B, Tumova S, Muraki K, Bruns A, Ludlow MJ, Sedo A, Hyman AJ, McKeown L, Young RS, Yuldasheva NY, Majeed Y, Wilson LA, Rode B, Bailey MA, Kim HR, Fu Z, Carter DA, Bilton J, Imrie H, Ajuh P, Dear TN, Cubbon RM, Kearney MT, Prasad RK, Evans PC, Ainscough JF, Beech DJ. Piezo1 integration of vascular architecture with physiological force. Nature 515: 279–282, 2014. doi:10.1038/nature13701. 25119035 PMC4230887

[35] Mendoza SA, Fang J, Gutterman DD, Wilcox DA, Bubolz AH, Li R, Suzuki M, Zhang DX. TRPV4-mediated endothelial Ca^2+^ influx and vasodilation in response to shear stress. Am J Physiol Heart Circ Physiol 298: H466–H476, 2010. doi:10.1152/ajpheart.00854.2009. 19966050 PMC2822567

[36] Osawa M, Masuda M, Kusano K, Fujiwara K. Evidence for a role of platelet endothelial cell adhesion molecule-1 in endothelial cell mechanosignal transduction: is it a mechanoresponsive molecule? J Cell Biol 158: 773–785, 2002. doi:10.1083/jcb.200205049. 12177047 PMC2174013

[37] Tzima E, Irani-Tehrani M, Kiosses WB, Dejana E, Schultz DA, Engelhardt B, Cao G, DeLisser H, Schwartz MA. A mechanosensory complex that mediates the endothelial cell response to fluid shear stress. Nature 437: 426–431, 2005. doi:10.1038/nature03952. 16163360

[38] Boldogh IR, Pon LA. Interactions of mitochondria with the actin cytoskeleton. Biochim Biophys Acta 1763: 450–462, 2006. doi:10.1016/j.bbamcr.2006.02.014. 16624426

[39] Kuznetsov AV, Javadov S, Guzun R, Grimm M, Saks V. Cytoskeleton and regulation of mitochondrial function: the role of beta-tubulin II. Front Physiol 4: 82, 2013. doi:10.3389/fphys.2013.00082. 23630499 PMC3631707

[40] Honerkamp-Smith AR, Woodhouse FG, Kantsler V, Goldstein RE. Membrane viscosity determined from shear-driven flow in giant vesicles. Phys Rev Lett 111: 038103, 2013. doi:10.1103/PhysRevLett.111.038103. 23909365

[41] Sebastian B, Favero T, Dittrich PS. The effects of shear force transmission across vesicle membranes. J Phys Chem Lett 8: 6128–6134, 2017. doi:10.1021/acs.jpclett.7b02676. 29190425 PMC6426246

[42] De Keulenaer GW, Chappell DC, Ishizaka N, Nerem RM, Alexander RW, Griendling KK. Oscillatory and steady laminar shear stress differentially affect human endothelial redox state: role of a superoxide-producing NADH oxidase. Circ Res 82: 1094–1101, 1998. doi:10.1161/01.res.82.10.1094. 9622162

[43] Hwang J, Ing MH, Salazar A, Lassegue B, Griendling K, Navab M, Sevanian A, Hsiai TK. Pulsatile versus oscillatory shear stress regulates NADPH oxidase subunit expression: implication for native LDL oxidation. Circ Res 93: 1225–1232, 2003. doi:10.1161/01.RES.0000104087.29395.66. 14593003 PMC4433384

[44] Hwang J, Saha A, Boo YC, Sorescu GP, McNally JS, Holland SM, Dikalov S, Giddens DP, Griendling KK, Harrison DG, Jo H. Oscillatory shear stress stimulates endothelial production of O_2_^-^ from p47^phox^-dependent NAD(P)H oxidases, leading to monocyte adhesion. J Biol Chem 278: 47291–47298, 2003. doi:10.1074/jbc.M305150200. 12958309

[45] Sorescu GP, Sykes M, Weiss D, Platt MO, Saha A, Hwang J, Boyd N, Boo YC, Vega JD, Taylor WR, Jo H. Bone morphogenic protein 4 produced in endothelial cells by oscillatory shear stress stimulates an inflammatory response. J Biol Chem 278: 31128–31135, 2003. doi:10.1074/jbc.M300703200. 12766166

[46] Takabe W, Jen N, Ai L, Hamilton R, Wang S, Holmes K, Dharbandi F, Khalsa B, Bressler S, Barr ML, Li R, Hsiai TK. Oscillatory shear stress induces mitochondrial superoxide production: implication of NADPH oxidase and c-Jun NH_2_-terminal kinase signaling. Antioxid Redox Signal 15: 1379–1388, 2011. doi:10.1089/ars.2010.3645. 20919940 PMC3144427

[47] Shanmugasundaram K, Nayak BK, Friedrichs WE, Kaushik D, Rodriguez R, Block K. NOX4 functions as a mitochondrial energetic sensor coupling cancer metabolic reprogramming to drug resistance. Nat Commun 8: 997, 2017. doi:10.1038/s41467-017-01106-1. 29051480 PMC5648812

[48] Sena LA, Chandel NS. Physiological roles of mitochondrial reactive oxygen species. Mol Cell 48: 158–167, 2012. doi:10.1016/j.molcel.2012.09.025. 23102266 PMC3484374

[49] Widlansky ME, Gutterman DD. Regulation of endothelial function by mitochondrial reactive oxygen species. Antioxid Redox Signal 15: 1517–1530, 2011. doi:10.1089/ars.2010.3642. 21194353 PMC3151425

[50] Zhang DX, Gutterman DD. Mitochondrial reactive oxygen species-mediated signaling in endothelial cells. Am J Physiol Heart Circ Physiol 292: H2023–H2031, 2007. doi:10.1152/ajpheart.01283.2006. 17237240

[51] Liu Y, Zhao H, Li H, Kalyanaraman B, Nicolosi AC, Gutterman DD. Mitochondrial sources of H_2_O_2_ generation play a key role in flow-mediated dilation in human coronary resistance arteries. Circ Res 93: 573–580, 2003. doi:10.1161/01.RES.0000091261.19387.AE. 12919951

[52] Chen K, Thomas SR, Albano A, Murphy MP, Keaney JF Jr. Mitochondrial function is required for hydrogen peroxide-induced growth factor receptor transactivation and downstream signaling. J Biol Chem 279: 35079–35086, 2004. doi:10.1074/jbc.M404859200. 15180991

[53] Wang Y, Zang QS, Liu Z, Wu Q, Maass D, Dulan G, Shaul PW, Melito L, Frantz DE, Kilgore JA, Williams NS, Terada LS, Nwariaku FE. Regulation of VEGF-induced endothelial cell migration by mitochondrial reactive oxygen species. Am J Physiol Cell Physiol 301: C695–C704, 2011. doi:10.1152/ajpcell.00322.2010. 21653897 PMC3174570

[54] Han Z, Varadharaj S, Giedt RJ, Zweier JL, Szeto HH, Alevriadou BR. Mitochondria-derived reactive oxygen species mediate heme oxygenase-1 expression in sheared endothelial cells. J Pharmacol Exp Ther 329: 94–101, 2009. doi:10.1124/jpet.108.145557. 19131585 PMC2670602

[55] Rhee SG. Redox signaling: hydrogen peroxide as intracellular messenger. Exp Mol Med 31: 53–59, 1999. doi:10.1038/emm.1999.9. 10410302

